# Inhibition of SIRT1 Impairs the Accumulation and Transcriptional Activity of HIF-1α Protein under Hypoxic Conditions

**DOI:** 10.1371/journal.pone.0033433

**Published:** 2012-03-30

**Authors:** Alexander Laemmle, Antje Lechleiter, Vincent Roh, Christa Schwarz, Simone Portmann, Cynthia Furer, Adrian Keogh, Mario P. Tschan, Daniel Candinas, Stephan A. Vorburger, Deborah Stroka

**Affiliations:** 1 Clinic of Visceral Surgery and Medicine, Visceral and Transplantation Surgery, University Hospital Bern and University of Bern, Bern, Switzerland; 2 Medical Oncology/Hematology, Department of Clinical Research, Inselspital, University Hospital Bern and University of Bern, Bern, Switzerland; Roswell Park Cancer Institute, United States of America

## Abstract

Sirtuins and hypoxia-inducible transcription factors (HIF) have well-established roles in regulating cellular responses to metabolic and oxidative stress. Recent reports have linked these two protein families by demonstrating that sirtuins can regulate the activity of HIF-1 and HIF-2. Here we investigated the role of SIRT1, a NAD+-dependent deacetylase, in the regulation of HIF-1 activity in hypoxic conditions. Our results show that in hepatocellular carcinoma (HCC) cell lines, hypoxia did not alter SIRT1 mRNA or protein expression, whereas it predictably led to the accumulation of HIF-1α and the up-regulation of its target genes. In hypoxic models *in vitro* and in *in vivo* models of systemic hypoxia and xenograft tumor growth, knockdown of SIRT1 protein with shRNA or inhibition of its activity with small molecule inhibitors impaired the accumulation of HIF-1α protein and the transcriptional increase of its target genes. In addition, endogenous SIRT1 and HIF-1α proteins co-immunoprecipitated and loss of SIRT1 activity led to a hyperacetylation of HIF-1α. Taken together, our data suggest that HIF-1α and SIRT1 proteins interact in HCC cells and that HIF-1α is a target of SIRT1 deacetylase activity. Moreover, SIRT1 is necessary for HIF-1α protein accumulation and activation of HIF-1 target genes under hypoxic conditions.

## Introduction

Silent information regulator 2 (Sir2) was initially identified in *Saccharomyces cerevisiae* as the first member of the highly conserved sirtuin family of proteins [1]. The mammalian homolog to Sir2 is SIRT1 and is the first of seven thus far described sirtuin family members (SIRT1–SIRT7). Sirtuin proteins are nicotinamide adenine dinucleotide (NAD+)-dependent deacetylases. Their dependency on NAD+ suggests that sirtuin activity serves as a sensor of the cytosolic ratio of NAD+/NADH and thus directly links sirtuin activity to the metabolic and cellular energy state of a cell [2], [3]. Since the discovery of their enzymatic activity, sirtuins have been implicated in many important physiological functions including gene silencing, apoptosis, energy maintenance and longevity, reviewed in [4].

SIRT proteins have different subcellular localizations and described functions. For example, SIRT1 and SIRT2 are found in both the nucleus and cytoplasm. SIRT1 regulate pathways in metabolism, inflammation and tumorigenesis and SIRT2 functions as a tubulin deacetylase [5]. SIRT3 and SIRT5 are localized in mitochondria and regulate metabolism and ammonia detoxification, respectively [6], [7]. Recently, it has been suggested that SIRT5 is also a NAD+-dependent demalonylase and desuccinylase [8]. SIRT4 is also located in the mitochondria and inhibits glutamate dehydrogenase activity [9]. SIRT6 is found in the nucleus and functions in maintaining genomic stability and glucose homeostasis [10], [11]. SIRT7 interacts with RNA polymerase I histones to promote Pol I-mediated rRNA transcription in the nucleolus [12].

SIRT1 is the most studied sirtuin family member, mainly due to its purported ability to promote longevity in yeast, worms, drosophila and mammals [13], [14], [15], [16]. However its ability to increase the life span of lower organisms has recently been called into question [17]. SIRT1 has also been suggested to have a critical role in tumorigenesis, however it is controversial whether SIRT1 is a tumor-suppressor or a tumor-promoter and in fact it is likely to be tumor-type specific [18]. SIRT1's deacetylase activity plays an important function in normal and malignant cellular processes by targeting histones, which results in a tighter chromatin structure and transcriptional repression [19]. Importantly, SIRT1 also modulates the stability and/or activation potential of a broad range of transcription factors, such as p53 [20], [21], FOXO [22], Ku70 [23], NF-κB [24], E2F1 [25] and PPARγ co-activator 1α (PGC-1α) [26] and as recently described the hypoxia-inducible transcription factors (HIF), HIF-1 [27] and HIF-2 [28].

HIF transcription factors are the key mediators of oxygen homeostasis under hypoxic conditions and they play a vital role in embryonic development, physiological responses and in disease pathologies. HIF heterodimers are composed of an oxygen-sensitive α-subunit and a constitutively expressed β-subunit. HIF-1 and HIF-2 are the best-characterized isoforms and are mainly regulated by posttranslational modifications of their α-subunit [29]. Specific prolyl hydroxylases (PHD), which depend on the substrates oxygen, Fe (II) and 2-oxoglutarate, target the α-subunit under normoxic conditions [30]. Hydroxylation of two proline residues (HIF-1α: P402 and P564 and HIF-2α: P405 and P531) within the oxygen-dependent degradation domain serve as a recognition site for the von Hippel-Lindau tumor suppressor (pVHL), a ubiquitin E3 ligase, which leads to the proteosomal degradation of the α-subunit [31], [32], [33]. In the absence of oxygen, PHDs are inactive and thereby HIFα proteins are stabilized. Accumulated HIFα protein translocates to the nucleus, forms a dimer with HIFβ and along with co-activators such as p300-CBP binds to hypoxia responsive elements (HRE) of target genes. HIF-1 and HIF-2 share the same consensus sequence G/ACGTG in their target genes [34] and have several common gene targets such as erythropoietin (EPO), vascular endothelial growth factor (VEGF) and glucose transporter 1 (GLUT-1) [35]. However, they also have unique transcriptional targets, HIF-1 is responsible for the regulation of many genes encoding enzymes involved in the glycolytic pathway, as well as the pro-apoptotic gene BCL2 adenovirus E1B-interacting protein 1 (BNIP3) and carbonic anhydrase 9 (CA9) [36], [37].

Recent reports have linked HIF and sirtuin families together by demonstrating that SIRT1, SIRT3 and SIRT6 can regulate the activity of HIF proteins [27], [28], [38], [39]. With regards to the interaction between SIRT1 and HIF proteins, Lim et al. demonstrated that SIRT1 binds to and deacetylates HIF-1α at lysine 674. This interaction blocks p300 recruitment to the promoter of HIF-1 target genes and thereby represses HIF-1 transcriptional activity [27]. Conflictingly, Dioum et al. have reported that SIRT1 does not target HIF-1α, rather it deacetylates HIF-2α, and their interaction promotes HIF-2 transcriptional activity [28]. In addition, because SIRT1 is a redox sensor and dependent on the metabolic status of the cell, its regulation by hypoxia has been a point of interest. In one report, SIRT1 is down regulated in hypoxic conditions due to decreased NAD+ levels [27], while in another report it is up regulated in a HIF-dependent manner [40]. From the current literature, one can conclude that the interaction between SIRT1 and HIF-1 and the resulting outcome of their interactions is still unclear.

Previously generated data from our lab were also in conflict with the above-mentioned studies. Therefore, here, we report our findings on the involvement of SIRT1 and HIF-1 in hypoxic conditions. First we looked at the influence of hypoxia on SIRT1 expression. We consistently observed that SIRT1 protein or mRNA was not modified in three different HCC cell lines cultured under hypoxic conditions. Abundant SIRT1 and HIF-1α protein are simultaneously expressed in hypoxic cells. From this observation and considering the reported negative association between SIRT1 and HIF-1 [21], we questioned whether inhibiting SIRT1 activity would thereby augment HIF-1 function under hypoxic conditions. Our data demonstrates that inhibition of SIRT1 activity reduces the hypoxia-induced transcriptional activity of HIF-1 gene targets and the accumulation of HIF-1α protein itself. We show that this regulation is relevant *in vivo* by demonstrating that SIRT1 inhibition leads to a decreased HIF-mediated response to systemic hypoxia. In addition, SIRT1 inhibition resulted in growth inhibition in a mouse xenograft tumor model of HCC. And finally we demonstrate that endogenous SIRT1 and HIF-1α co-immunoprecipitate and HIF-1 is a target of SIRT1 deacetylase activity. Taken together these data suggest that SIRT1 targets HIF-1α protein and that this interaction is required for HIF-1α activity in hypoxic conditions.

## Results

### SIRT1 and HIF-1α are simultaneously expressed in hypoxic cells

**Figure 1:**
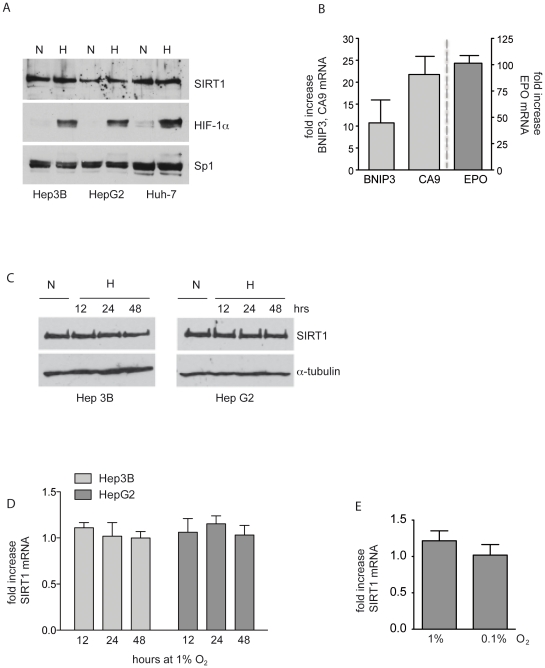
SIRT1 and HIF-1α are simultaneously expressed in hypoxic cells. **A.** Hep3B, HepG2 and Huh-7 cells were exposed to (N) normoxia (21% O_2_) or (H) hypoxia (1% O_2_) for 8 hours. SIRT1 and HIF-1α proteins were detected by Western blot. An antibody against Sp1 was used as a control for equal loading. A representative blot of 3 independently performed experiments is shown. **B.** Total RNA was isolated from Hep3B cells exposed to 1% O_2_ for 16 hours. RT-qPCR was performed and relative fold increase of target gene mRNA was calculated by comparing ΔCt values of cells cultured at 21% O_2_ to ΔCt values of cells exposed to 1% O_2_. Relative mRNA was increased 10.7±5-fold for BNIP3, 21.8±4-fold for CA9 and 101±17-fold. *Columns*, mean values from 3 independent experiments. *Bars*, ±SD. **C.** Hep3B and HepG2 cells were exposed to (N) normoxia (21% O_2_) or (H) hypoxia (1% O_2_) for 12, 24 and 48 hours. SIRT1 and α-tubulin proteins were detected in total cell lysates by Western blot. A representative blot of 6 independently performed experiments is shown. **D.** Total RNA was isolated from Hep3B cells exposed to 1% O_2_ for 12, 24 and 48 hours and ΔCt values of SIRT1 mRNA was compared to cells cultured at 21% O_2_ by RT-qPCR. *Columns*, mean values from 6 independent experiments. *Bars*, ±SD. **E.** Total RNA was isolated from Hep3B cells exposed to 1% O_2_ and 0.1% O_2_ for 24 hours and ΔCt values of SIRT1 mRNA was compared to cells cultured at 21% O_2_ by RT-qPCR. *Columns*, mean values from 6 independent experiments. *Bars*, ±SD. 1%O_2_.

HIF-1 is tightly regulated by oxygen availability and functions predominantly in hypoxic conditions. In order to demonstrate that SIRT1 is necessary for the accumulation of HIF-1α protein, we first verified that SIRT1 and HIF-1α proteins are co-expressed in hypoxic conditions. SIRT1 protein was strongly expressed in Hep3B, HepG2 and Huh7 HCC cells lines in normal culture conditions as well as in cells incubated at 1% O_2_ (Figure 1A). As predicted, HIF-1α protein was not detected in cells cultured at 21% O_2_ and was stabilized in cells incubated at 1% O_2_ (Figure 1A). Stabilization of HIF-1α protein led to the transcriptional increase of HIF-1 target genes BNIP3, CA9 as well as EPO, a target gene shared with HIF-2 (Figure 1B). To further verify that SIRT1 protein is not altered by hypoxia, HCC cells were exposed to 1% O_2_ for up to 48 hours. There was no change in SIRT1 protein or mRNA in cells exposed for 12, 24 or 48 hours to 1% O_2_ (Figure 1C & D). In addition, there was no significant change in SIRT1 mRNA in Hep3B cells exposed to more severe hypoxia of 0.1% O_2_ for 24 hours (Figure 1E). These data show that both SIRT1 and HIF-1α are present in cells under hypoxic conditions and HIF-1 is transcriptionally active. Moreover, unlike HIF-1α, SIRT1 expression is not tightly regulated by hypoxia in HCC cells.

### Inhibition of SIRT1 decreases HIF transcriptional activity and suppresses HIF-1α protein accumulation

**Figure 2:**
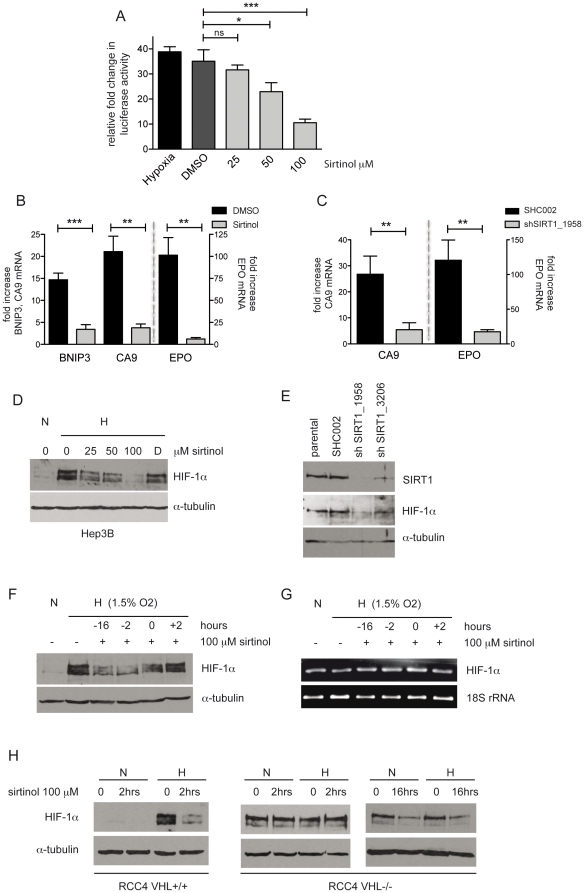
SIRT1 inhibition represses HIF-1 transcriptional activity and HIF-1α protein. **A.** Hep3B cells were co-transfected with luciferase reporter carrying multiple HREs and a renilla luciferase control plasmid. Twenty-four hours after the transfection, cells were treated with 25, 50 and 100 µM sirtinol and exposed to hypoxia for 24 hours. Dual luciferase activities were measured and firefly values were normalized by renilla values. Luciferase activity was reduced from 35-fold of DMSO-treated controls to 32-fold with 25 µM sirtinol, p = 0.2611; to 23-fold with 50 µM, *p = 0.0225 and to 10.5-fold with 100 µM ***p = 0.0009. *Columns*, mean of triplicates from one representative experiment (n = 3, independent experiments) *Bars*, ±SD. **B.** Hep3B cells were either treated with 100 µM sirtinol or an equivalent concentration of DMSO for 4 hours and then exposed to 1% O_2_ for 16 hours. Fold increase was calculated from ΔCt values of hypoxic cells to average ΔCt values of cells cultured at 21% O_2_ in DMSO. BNIP3 mRNA was reduced from 14±1.5 to 3.4±1-fold (***p = 0.0005), CA9 mRNA from 21±3.5 to 3.8±0.8-fold (**p = 0.0011) and EPO from 100±11.5 to 6.1±1.6-fold (**p = 0.0012) *Columns*, mean of 3 independent experiments; *bars*, ±SD. **C.** Hep3B cells were infected with lentiviral vectors containing shRNA sequences that target SIRT1 (shSIRT1_1958) or with a scrambled control (SHC002). Five days after transduction cells were exposed to hypoxia for 12 hours. The relative fold increase of mRNA in hypoxic cells was calculated compared to normoxic controls. CA9 mRNA reduced from 27- to 5.5-fold (**p = 0.008) and EPO from 120- to 18-fold (**p = 0.004). *Columns*, mean of 3 independent experiments; *bars*, ±SD. **D.** Hep3B cells were treated with 0, 25, 50 and 100 µM sirtinol or an equivalent concentration of DMSO (D) for 16 hours and then exposed to 21% O_2_ (N) or 1% O_2_ (H) for 4 hours. Whole cell lysates were analyzed by Western blot using antibodies against HIF-1α. α-tubulin was used as a loading control. A representative blot of 6 independently performed experiments is shown. **E.** Hep3B cells were infected with lentiviral vectors containing shRNAs targeting SIRT1 (shSIRT1_1958 and shSIRT1_3206) or with a scrambled negative control (SHC002). Five days after transduction, cells were exposed to hypoxia for 4 hours and SIRT1 and HIF-1α were analyzed by Western blot. Representative blot of 3 independently performed experiments. **F–G.** Hep3B cells were treated with 100 µM sirtinol for 16, 2 and 0 hours before and 2 hours after the exposure to hypoxia for a total of 4 hours. HIF-1α expression was analyzed by Western blot and by RT-qPCR. **H.** RCC4 VHL+/+ and RCC4 VHL−/− cells were pretreated with 100 µM sirtinol or DMSO for 2 or 16 hours, followed by 4 hours exposure to 21% O_2_ (N) or 1% O_2_ (H). Whole cell lysates were analyzed for HIF-1α by Western blot.

As demonstrated above, we observed a simultaneous expression of both SIRT1 and HIF-1α protein under hypoxic conditions. A recent report by Lim et al. has suggested that SIRT1 negatively regulates HIF-1 activity by suppressing the transcriptional regulation of its target genes [27]. Following the model proposed by their data, inhibition of SIRT1 should provoke an exaggerated HIF-mediated response. Therefore, we tested the effect of inhibiting SIRT1 on HIF-1 activity using sirtinol, a cell permeable specific inhibitor of SIRT deacetylase activity [41]. The IC_50_ value of sirtinol is 70 µM for SIRT1 activity; hence concentrations of 25, 50 and 100 µM were used in this study. First, we confirmed the functionality of sirtinol on SIRT1 deacetylase activity in HepG2 cells. HepG2 cells have abundant SIRT1 protein, as well as an intact p53 response to DNA damage. The acetylation of lysine 382 of p53 is modulated by SIRT1 [21]. We demonstrated that sirtinol had no detectable effect on the lysine 382 acetylation in the absence of DNA damage, whereas, sirtinol produced a time-dependent increase of acetylated p53 in cells treated with the DNA damaging agent, doxorubicin (Figure S1). These data confirm that sirtinol is an efficient inhibitor of SIRT1 activity *in vitro*. We next investigated the effect of inhibiting SIRT1 activity on HIF-mediated transcriptional activation. Cells were transfected with a HRE reporter construct that contains multiple HRE sites and is specifically induced by HIF proteins and thus represents a direct measurement of HIF activation [42]. Twenty-four hours after transfection, cells were treated with increasing doses of sirtinol and incubated 24 hours at 1% O_2_. Hypoxia increased reporter activity 38-fold and there was no significant change in cells treated with DMSO at a concentration equal to the amount needed to treat cells with 100 µM sirtinol. There was a dose-dependent decrease of HIF-1 transcriptional activity in cells treated with sirtinol. A significant reduction of reporter activity was observed with 50 µM and 100 µM sirtinol (Figure 2A). We next verified that inhibition of SIRT1 activity alters the transcriptional activity of HIF-1 target genes. Pretreatment with 100 µM sirtinol significantly reduced the hypoxic induction of specific HIF-1 targets BNIP3 and CA9 mRNA as well as EPO mRNA (Figure 2B). Although, sirtinol specifically inhibits SIRT1, it also affects other members of the sirtuin family such as SIRT2 with an IC_50_ value of 40 µM [41]. Therefore to verify that the effect of sirtinol on repressing HIF-mediated transcriptional activity is at least in part due to the inhibition of SIRT1, cells were infected with lentiviruses carrying shRNA sequences targeting SIRT1. Targeted disruption of SIRT1 with clone shSIRT1_1958 led to a nearly complete knockdown whereas, shSIRT1_3206 resulted in a partial knockdown of SIRT1 protein compared to parental and SHC002 controls (Figure 2E). Cells infected with lentiviruses expressing clone shSIRT1_1958 significantly reduced the hypoxic induction of CA9 and EPO mRNA (Figure 2C). These data demonstrate that inhibition of SIRT1 activity with a small molecule inhibitor and a genetic knockdown leads to a strong decrease of HIF-1-mediated transcriptional activity.

We next questioned, whether loss of HIF transcriptional activity by inhibiting SIRT1 was due to an effect on HIF-1α protein itself. Hep3B cells were incubated with sirtinol for 16 hours and then exposed to 1% O_2_ for 4 hours. HIF is mainly regulated by hydroxylation of its α-subunit, so as expected, in normoxic Hep3B cells HIF-1α protein was degraded and nearly undetectable, whereas it was efficiently stabilized under hypoxia. Interestingly, inhibition of SIRT1 activity with sirtinol led to a dose-dependent repression of HIF-1α protein accumulation (Figure 1D). Importantly, HIF-1α was detected by using an antibody that is specific for HIF-1α and does not cross react with HIF-2α [43]. To verify that the repressive effect on HIF-1α expression was due to SIRT1 inhibition, the experiment was repeated in Hep3B with a knockdown of SIRT1 expression. SIRT1 knockdown cells had an impaired ability to accumulate HIF-1α protein; the efficiency of SIRT1 knockdown correlated with the suppression of HIF-1α protein, with a stronger effect achieved in cells infected with lentivirus shSIRT1_1958 (Figure 2E).

To determine the kinetics of sirtinol-mediated HIF-1α protein repression, Hep3B cells were either incubated with sirtinol for 16, 2 and 0 hours before exposing them to hypoxia for 4 hours or sirtinol was added to the cells 2 hours after exposure to hypoxia. Sirtinol added at the onset of hypoxia inhibited the accumulation of HIF-1α protein. An even stronger repressive effect was observed when cells were pre-exposed to sirtinol for 2 or 16 hours. Addition of sirtinol to cells after two hours under hypoxia, resulted in no reduction of HIF-1α protein (Figure 2F). Using the same experimental setting, we checked whether SIRT1 inhibition impairs the transcription of HIF-1α. HIF-1α mRNA levels were not modified by sirtinol (Figure 2G). RT-qPCR showed similar results as equivalent ΔCt values were calculated for each time point and condition tested (data not shown). This analysis of HIF-1α mRNA, suggests that impairment of HIF-1α protein accumulation is not a result of an inhibition of its transcription.

The degradation of HIFα proteins is mediated by VHL. To determine whether the sirtinol-induced repression of HIF-1α protein is dependent on VHL, we used a VHL-deficient renal cell carcinoma, RCC4 VHL−/− and a VHL reconstituted, RCC4 VHL+/+ cell lines. RCC4 VHL−/− cells express HIF-1α protein constitutively, even under normoxic conditions [44], [45]. Exposure of RCC4 VHL+/+ cells to hypoxia for 4 hours, led to the induction of HIF-1α protein. Two hours of sirtinol pretreatment strongly inhibited hypoxia-induced HIF-1α accumulation in RCC4 VHL+/+ cells (Figure 2H). In RCC4 VHL−/− cells, sirtinol repressed HIF-1α protein under both normoxic and hypoxic conditions, thus demonstrating that sirtinol-mediated HIF-1α repression is independent of VHL (Figure 2H). Interestingly, RCC4 VHL−/− cells required a longer treatment time with sirtinol (16 hours) in order to decrease HIF-1α protein levels. The shorter incubation time (2 hours) with sirtinol was insufficient to observe an effect on HIF-1α protein (Figure 2H). This observation is consistent with our findings in Hep3B cells (Figure 2F), indicating that sirtinol-mediated repression of HIF-1α is due to a decrease of newly stabilized HIF-1α protein, rather than enhanced degradation of preformed (mature) HIF-1α.

To verify that the inhibition of SIRT1 represses HIF-1α in a cell independent manner, two additional cell lines were tested. Similar to the results obtained in Hep3B cells, sirtinol pretreatment decreased hypoxia-induced HIF-1α protein stabilization in HepG2 and Huh7 cells (Figure S2). Taken together, these results show that reducing SIRT1 protein expression or deacetylase activity impairs the ability of HIF-1α protein to accumulate under hypoxic conditions and to transcriptionally activate its target genes. Moreover, SIRT1 is not regulating HIF-1α at the transcriptional level but it is required for the post-translational stabilization and accumulation of HIF-1α protein.

### SIRT1 overexpression enhances hypoxic stabilization of HIF-1α protein

**Figure 3:**
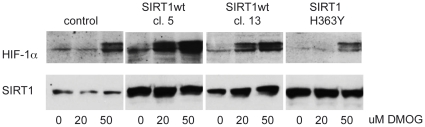
SIRT1 overexpression stabilizes HIF-1α protein. Hep3B cells were infected with lentiviruses expressing either SIRT1wt or mutated SIRT1 H363Y. Three days after transduction clones were selected and expanded. Hep3B cells with SIRT1wt clone 5 and clone 13 or SIRT1 H363Y lines were incubated with 0, 20 and 50 µM DMOG for 2 hours. Whole cell lysates were analyzed for SIRT1 and HIF-1α by Western blot. A representative blot of 3 independently performed experiments is shown.

The data presented above suggest that the inhibition of SIRT1 impairs the accumulation of HIF-1α protein under hypoxic conditions. We next tested if enhanced SIRT1 expression has a stabilizing effect on HIF-1α protein. Hep3B cells were infected with lentiviruses expressing SIRT1 wild type protein or SIRT1 H363Y protein, which is point-mutated in the catalytic domain of SIRT1 and therefore enzymatically inactive [46]. Clones of Hep3B cells overexpressing wild type or mutated SIRT1 were incubated with increasing concentrations of dimethyloxallyl glycine (DMOG) a competitive inhibitor of HIF PHDs used to stabilize HIF-1α under normoxic conditions. Cells overexpressing wild type SIRT1 showed a greater sensitivity to DMOG, as lower concentrations of DMOG were able to stabilize HIF-1α in SIRT1 wild type cells compared to SIRT1 mutated and control cells (Figure 3). Overexpression of SIRT1 showed a stronger effect on the accumulation of HIF-1α than SIRT1 H363Y, thus suggesting that SIRT1 deacetylase activity plays a role in the stabilization of HIF-1α.

### HIF-1α interacts with SIRT1 protein and is a target of its deacetylase activity

**Figure 4:**
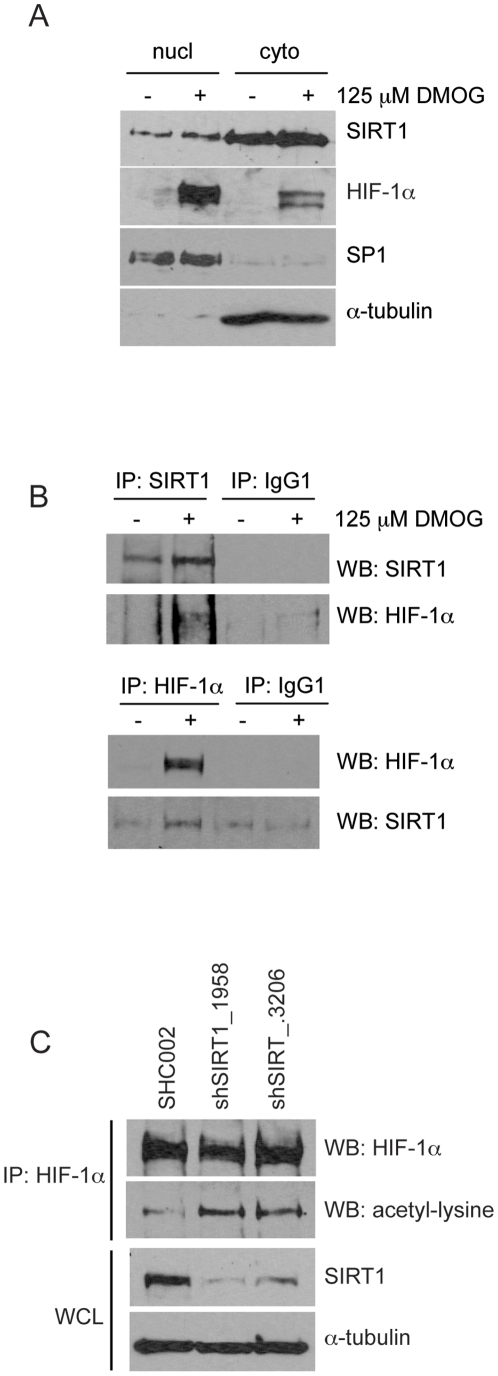
SIRT1 and HIF-1α co-immunoprecipitate. **A.** Hep3B cells were treated with 125 µM DMOG for 5 hours. Cytoplasmic and nuclear protein fractions were prepared. Twenty-five µg nuclear proteins and 50 µg cytoplasmic proteins were analyzed for SIRT1 and HIF-1α by Western blot. Sp1 was used as a nuclear fraction marker and α-tubulin for the cytoplasmic fraction. A representative blot of 3 independently performed experiments is shown. **B.** Cytoplasmic proteins (1250 µg) prepared in (A) were immunoprecipitated with a rabbit polyclonal SIRT1 antibody, chicken polyclonal HIF-1α antibody or a rabbit IgG1 control antibody. Immunoprecipitated proteins were subjected to Western blot analysis. A representative blot of 3 independently performed experiments is shown. **C.** SIRT1 inhibition leads to increased acetylation of HIF-1α. Hep3B cells were infected with lentiviral vectors containing shRNA against SIRT1 (shSIRT1_1958 or shSIRT1_3206) or scrambled shRNA (SHC002) as a negative control. Seven days post-infection, cells were incubated under hypoxia for 5 hours in the presence of 10 µM MG132. Whole cell lysates (2 mg) were immunoprecipitated with a mouse monoclonal HIF-1α antibody (2 µg) and precipitates were analyzed by Western blotting with a rabbit polyclonal anti-acetyl-lysine and a polyclonal chicken anti-HIF-1α antibody. Whole cell lysates (WCL: 100 µg) were used as input controls and were analyzed for SIRT1 and α-tubulin. A representative blot of 2 independently performed experiments is shown.

We next questioned whether HIF-1α is a target of SIRT1 deacetylase activity. There is contradictory literature on whether there is a direct interaction between HIF-1α and SIRT1 [27], [28]. Therefore, we tested if endogenous proteins co-immunoprecipitate in the cells used in this study. Hep3B cells were treated with DMOG for 5 hours to stabilize HIF-1α under normoxic conditions. SIRT1 protein was detected in the cytoplasmic and nuclear fraction. A strong HIF-1α signal was found in the nuclear fraction, and a lower signal was detected in the cytoplasmic fraction (Figure 4A). Using endogenously expressed cytoplasmic proteins, we observed that HIF-1α immunoprecipitated with extracts for SIRT1, and likewise, SIRT1 is detected in extracts immunoprecipitated for HIF-1α (Figure 4B). In summary, our data are in agreement with others, and suggest that SIRT1 and HIF-1α proteins physically interact [27], [39]. From this data we hypothesize that SIRT1 may exert its effects on HIF-1α protein stability through a physical interaction.

Having demonstrated that SIRT1 and HIF-1α physically interact in Hep3B cells, we investigated whether HIF-1α is a target of SIRT1 deacetylase activity. Hep3B cells were infected with lentiviruses containing shRNA against SIRT1 or SHC002 as negative control. As shown in Figure 2E, SIRT1 knockdown leads to a loss of HIF-1α protein. Therefore, to investigate changes in the acetylation level of HIF-1α protein in the SIRT1 knockdown cells, MG132 was added to avoid HIF-1α proteosomal degradation. HIF-1α proteins were immunoprecipitated then analyzed by Western blot using a polyclonal anti-acetyl-lysine antibody. There was a clear increase of acetylated HIF-1α protein in the SIRT1 knockdown cells compared to the SHC002-infected control (Figure 4C). From this experiment we can conclude that SIRT1 deacetylates HIF-1α protein, as knocking down of SIRT1 led to an increase of HIF-1α acetylation. We however cannot conclude that altering the acetylation state of HIF-1α affects its stabilization under hypoxic conditions.

### Cambinol blocks HIF-dependent gene regulation *in vivo*

**Figure 5:**
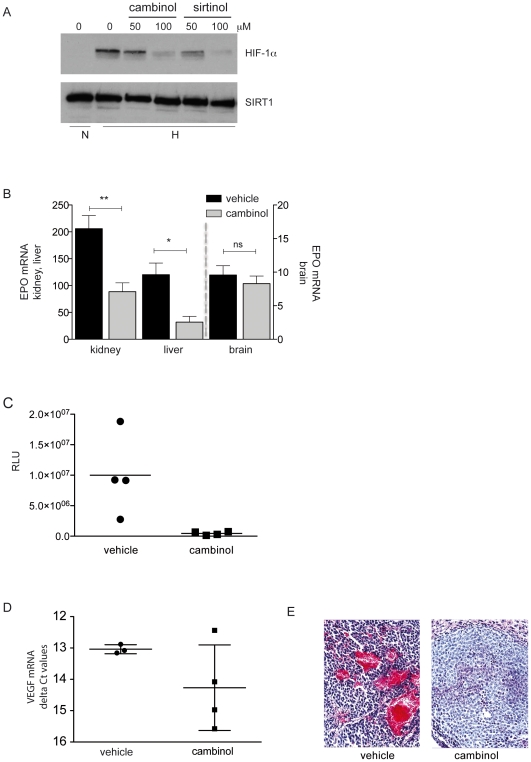
Inhibition of SIRT1 with cambinol impairs hypoxic response *in vivo*. **A.** HepG2 cells were treated with 50 and 100 µM sirtinol or cambinol or an equivalent concentration of DMSO (0) for 16 hours and then exposed to 21% O_2_ (N) or 1% O_2_ (H) for 4 hours. A representative blot of 2 independently performed experiments is shown. **B.** Wild type C57BL/6 mice were administered 100 mg/kg cambinol 2 hours prior to exposure to 6% O_2_ for 6 hours. Total RNA was isolated from liver, kidney and brain tissues. RT-qPCR was performed and ΔCt values of EPO mRNA of hypoxic mice treated with vehicle or cambinol were compared the average delta Ct values of vehicle-treated normoxic mice. The fold change of EPO mRNA was from 221±65 (control) to 88±48 (cambinol), **p = 0.0014 in the kidney and from 120±43 (control) to 21±10.6 (cambinol), *p = 0.01 in the liver and from 9.6±2.7 (control) to 8.3±2.3 (cambinol), p = 0.5036 in the brain. *Columns*, mean values from 4 independent experiments ±SD. **C.** Relative luciferase units (RLU) were measured as an indicator of tumor size in mice harboring intrahepatic luciferase-labeled HepG2 tumors. Mice treated with 100 mg/kg cambinol had an overall smaller tumor volume compared to vehicle treated controls. Dots are representative of individual animals, bars are the mean ±SD. **D.** Total RNA was isolated from HepG2 tumors. ΔCt values of VEGF mRNA in vehicle treated mice were compared to mice treated with cambinol by RT-qPCR. Dots are representative of individual animals and bars are the mean ±SD. **E.** Haematoxylin and eosin stain of excised tumors from mice treated with vehicle or cambinol.

We next tested if pharmacologically targeting SIRT1 can also influence HIF activity *in vivo*. Cambinol, a cell permeable β-naphthol compound inhibits the NAD+-dependent deacetylase activity of SIRT1 and SIRT2 (IC50 = 56 µM and 59 µM, respectively) and exhibits no inhibition against class I or II histone deacetylase activity [47]. Unlike sirtinol, cambinol can be used *in vivo* and was shown to effectively inhibit xenograft BCL6-expressing Burkitt lymphoma growth in mice [47]. In a first step, the suppressive effect of cambinol was tested and compared to sirtinol in HepG2 cells *in vitro* (Figure 5A). HepG2 cells are tumorigenic in immune deficient mice, therefore provide the opportunity to test the effect of SIRT1 inhibition in an HCC xenograft model. Inhibition of SIRT activity with cambinol led to a dose-dependent repression of HIF-1α protein accumulation in HepG2 cells *in vitro*. Cells treated with sirtinol were used for comparison (Figure 5A). We previously reported that in mice exposed to 6% oxygen HIF-1α protein accumulates in various tissues and activates HIF target genes [48]. Therefore, we tested if inhibition of SIRT1 represses a HIF-driven response *in vivo*. Mice were pre-treated with cambinol for 2 hours and then exposed to 6% oxygen for 6 hours. Analysis of mouse tissues showed that there was a significant decrease of EPO mRNA in the kidney and the liver in mice pre-treated with cambinol, whereas, pre-treatment with cambinol did not reduce EPO mRNA in the brain (Figure 5B). In HCC, HIF proteins play an important role in tumor progression and their expression is a poor prognostic indicator [49], [50]. Therefore to examine the effect of inhibiting SIRT1 on HIF expression and function in HCC, 0.5×10^6^ luciferase-labeled HepG2 cells were injected into the subcapsular space of the left liver lobe in immune deficient Rag2/common gamma-null mice. On day 8 after injection, intrahepatic tumors were visible by bioluminescent imaging. Starting on day 9, an i.p. injection of cambinol (100 mg/kg) or vehicle was administered daily, 5 times per week. A preliminary study verified the concentration of cambinol used had no toxic effect to the animals; they displayed no weight loss or increased levels of serum transaminases (ALT & AST) (data not shown). Animals were euthanized on day 30 due to sizeable tumor growth in the vehicle-treated group. Animals treated with cambinol had overall smaller tumors than vehicle treated controls (Figure 5C). Analysis of tumor tissue at time of excision revealed lower mRNA levels of the HIF target gene and pro-angiogenesis factor, VEGF in cambinol treated mice (Figure 5D). Histological examination of the tumors of cambinol treated animals showed less vascular density and intratumoral hemorrhage (Figure 5E). Taken together, these *in vivo* observations support our *in vitro* data and further demonstrate that loss of SIRT1 activity impairs HIF-mediated responses to hypoxia.

## Discussion

SIRT1 and HIF independently regulate key metabolic pathways important in biological processes, such as tumorigenesis and aging, therefore, a clear understanding of the interaction between these two protein families is of high scientific and clinical interest. There are discrepancies present in the current literature regarding the interaction and consequence of SIRT1 on HIF-1 activity. Here we present our findings that suggest SIRT1 is necessary for the accumulation of HIF-1α protein and therefore is a positive regulator of HIF-1 transcriptional activity.

We showed that HCC cells have high SIRT1 protein expression and that there is a simultaneous expression of both HIF-1α and SIRT1 proteins under hypoxic conditions. SIRT1 protein or mRNA levels were not altered in cells under hypoxic conditions for up to 48 hours. Our findings in human HCC cells are similar to those reported in adult rat cardiac myocytes in which SIRT1 protein levels were not increased by hypoxia alone. However in cardiac myocytes, SIRT1 was strongly increased in cells exposed to repetitive cycles of hypoxia/re-oxygenation [51]. Nevertheless, these data are not in agreement with studies that report hypoxia up-regulates SIRT1 in a HIF-dependent manner [40] or that hypoxia down-regulates SIRT1 due to decreased NAD+ levels [27].

Our observation that abundant SIRT1 and HIF-1α proteins are expressed simultaneously in hypoxic HCC cells is important as it does not correspond with the proposed model of Lim et al., in which they suggest that SIRT1 deacetylates HIF-1 and impairs HIF-1 transcriptional activity [27]. We observe a robust transcriptional increase of HIF-1 target genes in hypoxic cells that possess high endogenous levels of SIRT1 protein. Moreover, if SIRT1 were a negative regulator of HIF-1α, we would expect an enhanced transcriptional response of HIF-1 target genes by inhibition of SIRT1 protein. Here we report the opposite effect; the transcriptional activity of HIF target genes was consistently impaired with the inhibition of SIRT1. Inhibition of SIRT1 activity with genetic knockdown or small molecule inhibitors repressed the up-regulation of HIF-1 target genes *in vitro*. These results were substantiated *in vivo* with mouse models of systemic hypoxia and HCC tumor xenografts. Dioum et al. also proposed that SIRT1 positively regulates cellular responses to hypoxia, albeit in a manner dependent on HIF-2 [16]. Lim et al. overcame the discrepancies in their data by demonstrating that SIRT1 facilitated the transcriptional activity of HIF-2α, whereas it repressed HIF-1α activity [17]. Our data show that SIRT1 is necessary for the hypoxic up-regulation of specific HIF-1 target genes, CA9 and BNIP3; a HIF-1-mediated increase of their mRNA was impaired with SIRT1 inhibition. Moreover, we demonstrate that SIRT1 is necessary for the accumulation of HIF-1α protein itself, using an antibody that is specific for HIF-1α and does not cross-react with HIF-2α [32]. HIF-1α did not accumulate in cells treated for at least 2 hours with sirtinol or cambinol before exposure to hypoxia or in cells with knocked down SIRT1 protein expression. Rane et al. also reported that knockdown of SIRT1 results in a loss of HIF-1α protein expression in cardiac myocytes, although the effect was more pronounced in cells exposed to hypoxia/re-oxygenation than hypoxia alone [51]. Taken together, our data clearly suggests that SIRT1 positively regulates the transcriptional activity of HIF-1. To address the conflicting reports on the regulation of HIF-1α by SIRT1, experimental conditions and cell or tissue type specificities may need to be taken in consideration.

The mechanism of how SIRT1 inhibition impairs the accumulation of HIF-1α protein is still unclear. Our data, in agreement with others, show an endogenous interaction between SIRT1 and HIF-1α [27], [39]. Lim et al. identified lysine 674 in HIF-1α as a target of SIRT1 deacetylase activity [27]. In agreement with their data, we showed that inhibiting SIRT1 led to an increased acetylation of HIF-1α protein. However, we cannot conclude that altering the acetylation state of HIF-1α directly influences its stability. Conflicting data regarding the acetylation of HIF-1α have been reported [52]. In a yeast two-hybrid assay, interaction of HIF-1α with an acetyltransferase termed mouse ARD1 (mARD1), was shown to enhance acetylation of a specific lysine residue (K532) within the ODD of HIF-1α. The same group described enhanced binding of VHL to acetylated HIF-1α, thus leading to an increase in its proteosomal degradation [53]. However, it was shown in several other studies, that the human variant of ARD1, hARD1 does not acetylate HIF-1α [54], [55]. In addition, several reports present different mechanisms in which class I and class II histone deacetylase enzymes regulate the stabilization and transcriptional activation of HIF-1α [52], [56].

The complexity of the regulation of HIF activity is becoming more apparent. SIRT1 could be in part functioning by targeting enzymes regulating HIF activity. PHD2 is the main PHD responsible for the hydroxylation of HIF-1α under normoxic conditions [57]. SIRT1 was shown to down-regulate PHD2 through its deacetylase function [51]. Inhibition of SIRT1 deacetylase activity could result in higher PHD2 activity leading to the loss of accumulation of HIF-1α. In addition, new proteins such as pyruvate kinase M2 (PKM2) were described as being required for HIF-1 transactivation activity [58]. PKM2 interacts directly with HIF-1α and promotes transactivation of HIF-1 target genes by enhancing HIF-1 binding and p300 recruitment to hypoxia response elements. PKM2 activity is regulated by post-translational modifications such as hydroxylation [58], tyrosine phosphorylation [59], sumoylation [60] and lysine acetylation [61]. On could hypothesize that sirtuins could exert an additional level of control over HIF proteins by the post-translational modification of PKM2.

SIRT1 and HIF-1 are both highly conserved and constitutively expressed proteins that are positive factors needed to resolve metabolic and oxidative stress. In addition, both are required for normal tissue development [62] and are both targets for post-transcriptional regulation by the microRNA, miR-119a-5p [51]. Therefore, as suggested by our findings, it seems plausible that their interaction should facilitate, not hinder each other's function for normal tissue homeostasis. Although SIRT1 and HIF-1 are vital for the maintenance of healthy tissue, their expression may also have undesirable consequences in a malignant environment. Our observation that SIRT1 is highly expressed in HCC cell lines is consistent with that of others who reported that SIRT1 is overexpressed in liver, colon, breast, and prostate cancers and squamous cell carcinomas [63], [64]. Cancer cells have the ability to hijack cellular processes that can promote their survival under harsh conditions that exist in a tumor microenvironment. SIRT1 overexpression could provide tumor cells a survival advantage. Transient overexpression of SIRT1 was shown to be sufficient to stimulate basal rates of autophagy, which is used by cancer cells to help them survive under stressful tumor microenvironment conditions [65]. In this context, the inhibition of SIRT1 is becoming a novel approach for the development of new treatment strategies for some cancers. However, many of the available sirtuin inhibitors have limited potency and isoform specificity. This has prompted the development of novel inhibitors that can distinguish between sirtuin family members to better target the desired effector function [66]. Studies reporting that sirtuins can influence the activity and function of HIF transcription factors are of high interest, since HIF proteins are also frequently overexpressed in cancers, are driving force in many steps of cancer progression and are a negative predictors for patient outcome (reviewed in [67]). As suggested by our data, an additional benefit of targeting SIRT1 would be the inhibition of HIF-1 activity. However, as introduced earlier, SIRT1 targets the activity of many other transcription factors and co-activators. An undesirable effect of inhibiting SIRT1 activity could be obtained if, for example, NF-κB is constitutively activated in the targeted cancer cells. SIRT1 suppresses NF-κB signaling [24], [68] and release of this suppression could stimulate cancer cell proliferation, inhibit apoptosis and increase angiogenesis and metastasis [69].

Interestingly, SIRT1 is not the only sirtuin family member shown to regulate HIF-1 function. SIRT6 expression interferes with HIF-1-mediated transcriptional activity by interfering at the promoter of target genes [38]. SIRT3, a mitochondrial deacetylase functions as a tumor suppressor protein by its ability to inhibit the generation of reactive oxygen species (ROS) [70]. SIRT3 regulates HIF-1 by maintaining low levels of ROS allowing PHD to be active, which results in the degradation of the α-subunit. With loss of SIRT3 expression cellular ROS levels increase, reducing PHD activity, thus leading to an increase of HIF-1α expression [39]. In agreement with the findings described above, we observe lower SIRT3 and SIRT6 mRNA expression in 5 out of 7 liver cancer cells lines tested and compared to normal human hepatocytes (unpublished observation). These observations provide additional arguments for the necessity of potent sirtuin activators and inhibitors that can distinguish between the different family members.

Understanding the connection between sirtuin and HIF proteins is complex and the current literature is in part, contradictory. Our data add information to help understand the interaction between SIRT1 and HIF-1 in order to gain more insight of their intricate association in vivo and in the progression of aging and tumorigenesis.

## Materials and Methods

### Cell culture

Human HCC cell lines, Hep3B and HepG2 cells were purchased from ATCC (LCG Standards) and Huh7 cells were given by J-F. Dufour (University of Bern, Switzerland) and originally obtained from the Japanese Collection of Research Bioresources (JCRB). The VHL-deficient renal cell carcinoma cell line, RCC4 VHL−/− and RCC4 VHL+/+ cells [33] were obtained from G. Camenisch (University of Zürich, Switzerland). Cell lines were cultured in DMEM medium with 10% fetal bovine serum, 100 U/ml penicillin and 100 µg/ml streptomycin (Life Technology). Cells were cultured under normal oxygen conditions of 21% O_2_, 5% CO_2_ at 37°C in a humidified incubator or hypoxic conditions in a microaerophilic system (Ruskinn, Biotrace International, UK) at 1% or 0.1% O_2_ and 5% CO_2_ humidified environment.

### Chemicals and reagents

Dimethyloxaloylglycine (DMOG) and sirtinol were purchased from Alexis Biochemicals (Lausen, Switzerland). The proteasome inhibitor MG132 (Z-LLL-CHO) and cambinol was purchased from Sigma-Aldrich (Basel, Switzerland). All chemicals were dissolved in DMSO as stock solutions (DMOG: 100 µmol/L; sirtinol: 50 µmol/L; cambinol: 50 µmol/L; MG132: 25 µmol/L) and diluted with cell culture medium to obtain final concentrations. In all experiments, the necessary amount of DMSO was added to all control conditions to reach the final concentration in the media as the compound treated cells.

### Western blotting

Western blots were performed according to standard protocols. Briefly, total cell lysates were prepared by direct lysis in modified RIPA buffer, proteins were separated by SDS-PAGE and transferred to nitrocellulose membranes. Membranes were blocked in 5% non-fat dry milk and incubated with primary antibodies overnight at 4°C. HRP-conjugated secondary antibodies were incubated for 1 hour at room temperature and membranes were developed with enhanced chemiluminescence (Western lightening Plus-ECL, Perkin Elmer). Primary antibodies used were rabbit polyclonal anti-SIRT1 (MW: ∼120 kDa), mouse monoclonal anti-α-tubulin (MW: ∼55 kDa) and rabbit polyclonal anti-Sp1 (MW:∼100 kDa) all from Santa Cruz (Santa Cruz, USA), rabbit polyclonal anti-acetyl-lysine from Cell Signaling (Allschwil, Switzerland), mouse monoclonal anti-HIF-1α (MW:∼115 kDa) from Alexis Biochemicals (Lausen, Switzerland). Chicken polyclonal anti-HIF-1α (MW:∼115 kDa) antibody was a courtesy by M. Gassmann (University of Zurich, Switzerland) [43]. HRP-conjugated secondary antibodies used were goat anti-rabbit (Dako, Baar, Switzerland), goat anti-mouse (Perbio Science S.A, Lausanne, Switzerland) and rabbit anti-chicken (Promega, Dubendorf, Switzerland).

### RNA extraction and quantitative RT-PCR

Total RNA was isolated using Trizol Reagent following the manufacturer's protocol (Life Technologies). cDNA was synthesized using Omniscript RT Kit (QIagen). Levels of mRNA were analyzed by quantitated RT-qPCR. Human probes for 18S rRNA (4310893E), HIF-1α (Hs00153153_m1), EPO (Hs00171267_m1), CA9 (Hs00154208_m1), SIRT1 (Hs00202021_m1), BNIP3 (Hs00969293_m1), VEGF (Hs00173626_m1) and mouse EPO (Mm01202755_m1) were obtained from ABI (Applied Biosystems, Rotkreuz, Switzerland). Relative changes in mRNA were calculated with the ΔΔCt method. Ct values of target gene expression (TG) was calculated relative to a reference gene control (RG) using the following formula ΔCt_TG_ = Ct_TG_−Ct_RG_. Experimental groups (TG) are normalized to control group (CG): ΔΔCt = ΔCt_TG_−ΔCt_CG_, and fold increase = 2^−ΔΔCt^.

### Immunoprecipitation

For cytoplasmic and nuclear protein fractionation cells were lysed with lysis buffer [10 mmol/L Hepes (pH 7.9); 10 mmol/L KCL; 0.1 mmol/L EDTA; 0.1 mmol/L EGTA; 1 mmol/L DDT; phosphatase inhibitors (Na_3_VO_4_, NaF, PMSF); a protease inhibitor cocktail (Sigma) and 0.5% NP-40]. The lysates were centrifuged at 14,000 rpm and the supernatant was transferred in a new tube (cytoplasmic fraction). For the extraction of nuclear proteins, cell pellet was re-suspended in 20 mmol/L Hepes, pH 7.9; 400 mmol/L KCL for 15 minutes on ice. Extracts were centrifuged for 5 min at 2000 rpm and the supernatant was kept (nuclear fraction). For the immunoprecipitation of SIRT1, cytoplasmic proteins (1250 µg) and nuclear extracts (250 µg) were incubated with 2 µg and 1 µg of rabbit polyclonal anti-SIRT1 antibody, respectively (Santa Cruz Biotechnology) or similar amounts of rabbit IgG1 control antibody overnight at 4°C. The same procedure was performed for immunoprecipitation of HIF-1α with a mouse monoclonal anti HIF-1α antibody (Alexis Biochemicals) or mouse IgG1 control antibody. The lysates were then incubated for a further hour at 4°C together with 50 µl (cytoplasmic fraction) or 30 µl (nuclear fraction) of Protein G magnetic microbeads (Miltenyi Biotec, Bergisch Gladbach, Germany) and loaded on μcolumns (Miltenyi Biotec) according to the manufacturer's protocol. Beads were first extensively washed with lysis buffer (composed of 2/3 of lysis buffer and 1/3 of nuclear extraction buffer) and then boiled with Laemmli sample buffer for five minutes and analyzed by SDS-PAGE as described before. To check the acetylation level of HIF-1α, cells were lysed with a lysis buffer containing 1% Triton-X 100, 0.5% NP-40, 10 mmol/L Tris, 150 mmol/L NaCl, 1 mmol/L EDTA, 1 mmol/L EGTA, phosphatase inhibitors (Na_3_VO_4_, NaF, ZnCl_2_) and a protease inhibitor cocktail (Sigma). From each sample, 2 mg of protein were incubated with 2 µg of mouse monoclonal HIF-1α antibody overnight at 4°C. Five percent of whole cell lysates were saved as input controls. For immunoprecipitation of HIF-1α the above described procedure was performed and samples were analyzed by SDS-PAGE.

### Plasmids, transfections and reporter gene assay

Three tandem repeats of the HIF-1 binding site from the transferrin promoter in the pGL3 luciferase vector (Promega). A HBS is part of the hypoxia-responsive element (HRE) of HIF target genes. pGL3-HRE plasmid was co-transfected with Renilla luciferase control plasmid (pRL-TK) with Effectene transfection reagent according to the manufacturer's protocol (Qiagen). Twenty-four hours after co-transfection, cells were treated with sirtinol and incubated under hypoxia for 24 hours. Absolute luminescence was measured according to the Dual-Luciferase® Reporter Assay protocol (Promega). The firefly luciferase values were normalized to the corresponding renilla luciferase control values.

### Lentiviral vectors expressing shRNAs and SIRT1 and SIRT1 H363Y

Lentivirus production, titer determination and transduction were carried out as previously described [71]. Two shRNA against SIRT1 (clone 1, ID: NM_012238.3-1958s1c1 termed shSIRT1_1958 and clone 2, ID: NM_012238.3-3206s1c1 termed shSIRT1_3206) and a non-target shRNA control vector (SHC002) were obtained from Sigma (Basel, Switzerland). Cells were transduced overnight and then selected in 1.5 µg/ml puromycin for 3 to 5 days before experiments were performed. SIRT1 wt (wildtype) and SIRT1 H363Y (point-mutated) plasmids [46] were a kind gift from Dr. Michael Potente (University of Frankfurt, Frankfurt, Germany).

### *In vivo* models

Experiments were performed in strict accordance with Swiss Federal Veterinary Office article 18 Animal Welfare Act, article 141 Animal Welfare Ordinance and article 30 Animal Experimentation Ordinance. All protocols were approved by Bernese cantonal authorities, LANAT Amt für Landwirtschaft und Natur Veterinärdienst (VeD), Permit Nrs: 79/07 and 87/09. All surgery was performed under anesthesia and animals received post-surgical analgesia to minimize any potential suffering or discomfort.

An *in vivo* systemic, normobaric hypoxia model was performed as previously described [48]. For HCC orthotopic xenograft studies 0.5×10^6^ luciferase-labelled HepG2 cells were injected into the subcapsular space of the left liver lobe of 6–8 week old female Rag2 common gamma-null mice. Tumor growth was monitored with bioluminescent imaging using CCD camera (NightOWL, Berthold Technologies, Switzerland). Tumors were excised and embedded in paraffin or snap frozen in liquid nitrogen for further analysis.

### Statistical Analysis

Unless otherwise indicated, all graphs represent mean values ± standard deviation (SD). Statistics were performed using unpaired Students T-test and graphs made using GraphPad Prism software.

## Supporting Information

Figure S1 **Inhibition of SIRT1 increases DNA-damage induced acetylated p53.** HepG2 cells were pre-treated with 100 µM sirtinol or an equal concentration of DMSO for 4 hours and then incubated with 17 µM doxorubicin for 0.5, 1, 2, 6 and 18 hours. Total cell lysates were analyzed by Western blot. Cells treated with sirtinol displayed more acetylated p53 after DNA damage at earlier time points compared to DMSO-treated controls.
(TIF)

Figure S2 **Inhibition of SIRT1 impairs HIF-1α protein accumulation in HepG2 and Huh7 cell lines.** HepG2 and Huh7 cells were treated with 100 µM sirtinol or an equivalent concentration of DMSO (D) for 16 hours and then exposed to 21% O2 (N) or 1% O2 (H) for 4 hours. Whole cell lysates were analyzed by Western blot using antibodies against HIF-1α. α-tubulin was used as a loading control. A representative blot of 3 independently performed experiments is shown.
(TIF)
