# Supplementary figures and images for: Inhibition of SIRT1 Impairs the Accumulation and Transcriptional Activity of HIF-1α Protein under Hypoxic Conditions
Source: PLoS One. 2012 Mar 30;7(3):e33433. doi: 10.1371/journal.pone.0033433 (PMC3316573; doi:10.1371/journal.pone.0033433)

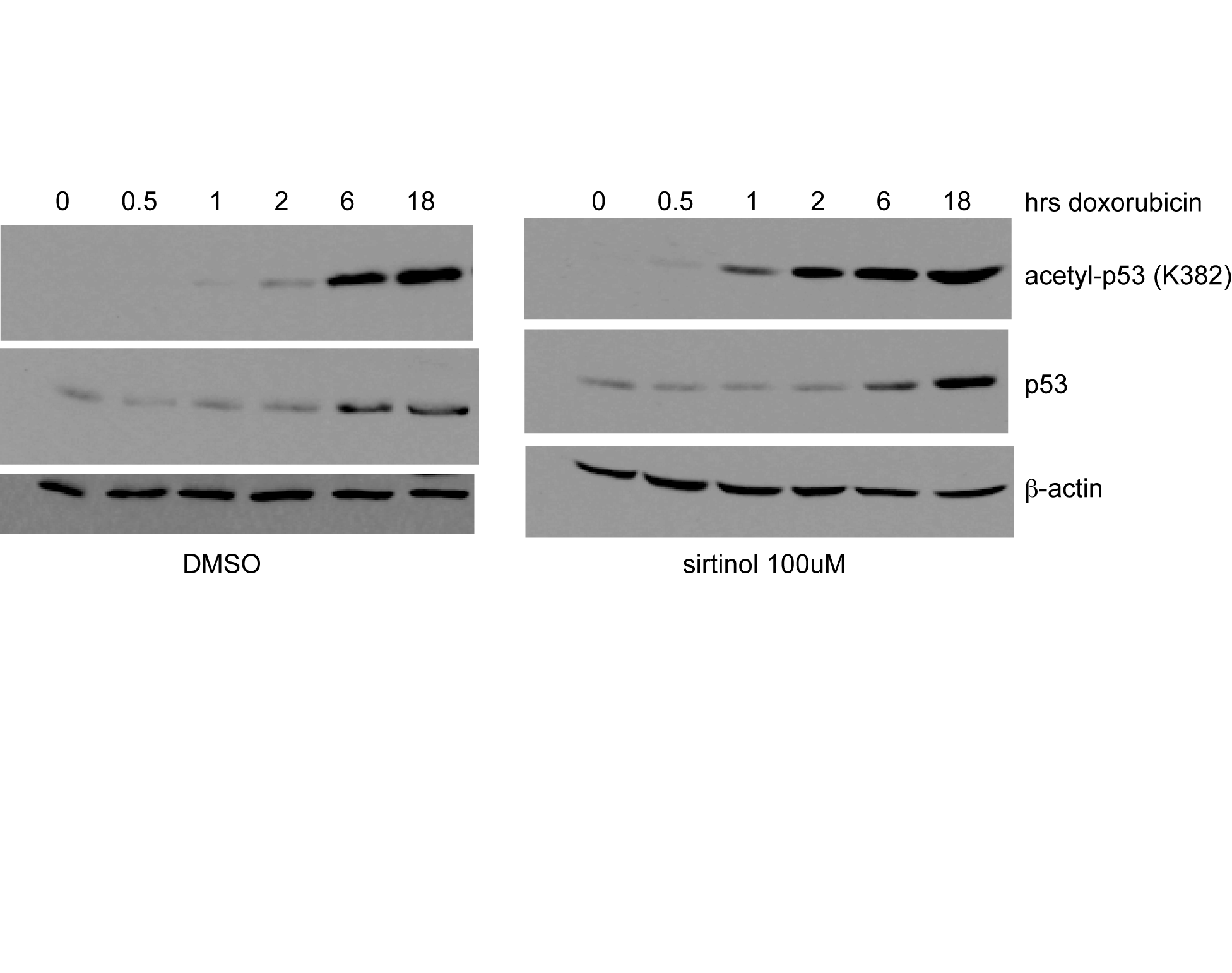

Supplement: Figure S1 — Inhibition of SIRT1 increases DNA-damage induced acetylated p53. HepG2 cells were pre-treated with 100 µM sirtinol or an equal concentration of DMSO for 4 hours and then incubated with 17 µM doxorubicin for 0.5, 1, 2, 6 and 18 hours. Total cell lysates were analyzed by Western blot. Cells treated with sirtinol displayed more acetylated p53 after DNA damage at earlier time points compared to DMSO-treated controls. (TIF) [file pone.0033433.s001.tif]

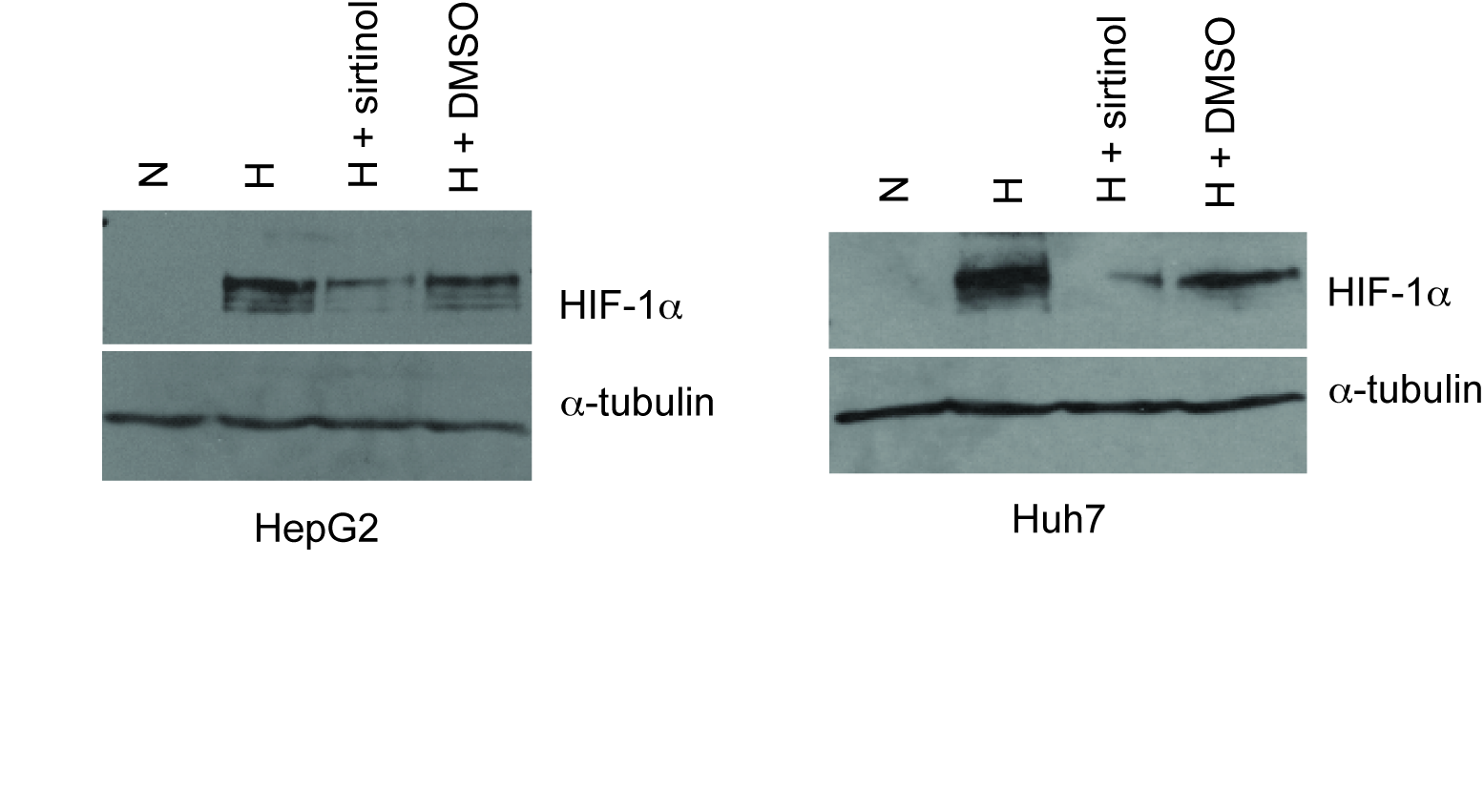

Supplement: Figure S2 — Inhibition of SIRT1 impairs HIF-1α protein accumulation in HepG2 and Huh7 cell lines. HepG2 and Huh7 cells were treated with 100 µM sirtinol or an equivalent concentration of DMSO (D) for 16 hours and then exposed to 21% O2 (N) or 1% O2 (H) for 4 hours. Whole cell lysates were analyzed by Western blot using antibodies against HIF-1α. α-tubulin was used as a loading control. A representative blot of 3 independently performed experiments is shown. (TIF) [file pone.0033433.s002.tif]
